# Protein receptor-independent plasma membrane remodeling by HAMLET: a tumoricidal protein-lipid complex

**DOI:** 10.1038/srep16432

**Published:** 2015-11-12

**Authors:** Aftab Nadeem, Jeremy Sanborn, Douglas L. Gettel, Ho C. S. James, Anna Rydström, Viviane N. Ngassam, Thomas Kjær Klausen, Stine Falsig Pedersen, Matti Lam, Atul N. Parikh, Catharina Svanborg

**Affiliations:** 1 Department of Microbiology, Immunology and Glycobiology (MIG), Institute of Laboratory Medicine, Lund University, S-223 62 Lund, Sweden; 2 Departments of Applied Science, Biomedical Engineering, and Chemical Engineering & Materials Science, University of California, Davis, CA 95616 USA; 3 Centre for Biomimetic Sensor Science, School of Materials Science and Engineering, Nanyang Technological University, 50 Nanyang Drive, 637553, Singapore; 4 Department of Biology, University of Copenhagen, Universitetsparken 13, 2100, Copenhagen, Denmark

## Abstract

A central tenet of signal transduction in eukaryotic cells is that extra-cellular ligands activate specific cell surface receptors, which orchestrate downstream responses. This ‘’protein-centric” view is increasingly challenged by evidence for the involvement of specialized membrane domains in signal transduction. Here, we propose that membrane perturbation may serve as an alternative mechanism to activate a conserved cell-death program in cancer cells. This view emerges from the extraordinary manner in which HAMLET (Human Alpha-lactalbumin Made LEthal to Tumor cells) kills a wide range of tumor cells *in vitro* and demonstrates therapeutic efficacy and selectivity in cancer models and clinical studies. We identify a ‘’receptor independent” transformation of vesicular motifs in model membranes, which is paralleled by gross remodeling of tumor cell membranes. Furthermore, we find that HAMLET accumulates within these *de novo* membrane conformations and define membrane blebs as cellular compartments for direct interactions of HAMLET with essential target proteins such as the Ras family of GTPases. Finally, we demonstrate lower sensitivity of healthy cell membranes to HAMLET challenge. These features suggest that HAMLET-induced curvature-dependent membrane conformations serve as surrogate receptors for initiating signal transduction cascades, ultimately leading to cell death.

Lipids alone suffice to produce closed, flexible bilayer membranes that isolate cellular interiors from the extra-cellular space^1^. A unique combination of material properties characterizes membrane elasticity^2^, including large volume compressibility (10^9^ to 10^10^ N/m^2^), area expansion (10^2^ to 10^3^ mN/m) and low bending rigidities (10^−19^ Nm). As a result, the vesicular membrane is highly resistant to compression and expansion in surface area but highly susceptible to bending-mediated deformations and shape transformations^3^,^4^. In the context of a living cell, these gross morphological shape changes are not a passive consequence of cellular activity, but present *de novo* membrane conformations, which are actively modulated during a variety of cellular responses^5^,^6^. Despite the widespread appreciation of membrane conformations in general, how they affect ligand binding, such as during signal transduction, is largely unknown.

Lipid membranes can potentially provide the cell with alternative mechanisms for sensing and binding external ligands. Ligand binding can modulate membrane properties, such as through morphological remodeling and/or spatial compositional reorganization, which can be translated by the cell into the biochemical language of signal transduction. For example, the binding of BAR domain superfamily and the COPII coat proteins mold membrane domains into highly specific, well-defined shapes and sizes^7^,^8^,^9^,^10^,^11^,^12^. BAR domains perform diverse functions, including *de novo* generation and sensing of membrane curvature for recruitment of cytosolic factors to differently shaped membrane structures, tenth of nanometer in size. COPII coat proteins favor nanometer-sized vesicles (60 nm–300 nm), depending on the types of recruited coat proteins and the availability of different cargo proteins^13^. In addition, exogenous ligands such as Shiga toxin (STX) may introduce sub-micrometer membrane invaginations, characterized by negative curvature in lipid organization, thereby deforming cell membranes^14^,^15^. In this case, one STX B-subunit molecule binds up to fifteen extracellular oligosaccharide receptor domains presented by the Gb_3_ membrane glycosphingolipids^16^.

The HAMLET complex is derived from human α-lactalbumin (HLA) – a major protein component in breast milk (2 mg/ml), which is a coenzyme in lactose synthase. In HLA, a large α-helical domain is separated from a small β-sheet domain by a deep cleft, which contains a disulfide bridge coordinating a Ca^2+^ binding loop at the junction of the two domains^17^,^18^,^19^. When the strongly bound Ca^2+^ is released, HLA adopts a partially unfolded conformation and this conformer binds a discrete number of oleic acid molecules, predominantly in the deprotonated oleate form, producing the long-lived, kinetically trapped HAMLET complex^20^. HAMLET’s mechanism of tumoricidal action is thought to involve a multi-step process. Shared by a broad variety of tumor cells, the process involves (1) cell-surface binding^21^; (2) ion flux activation^22^ (3) cytosolic uptake leading to intracellular interactions with kinases and GTPases^23^, resulting in broad inhibition of multiple signaling pathways and (4) eventual translocation to the cell nucleus, where HAMLET interacts with histones and disrupts the chromatin structure^24^.

This study examined if the insertion of HAMLET alone can achieve membrane remodeling, without engaging specific cell-surface receptors. We present evidence that HAMLET transforms the vesicular motif in model membranes into a dense tangle of tubules and grossly remodels plasma membranes of tumor cells, generating a positive membrane curvature, culminating in membrane protrusions and blebs. We also show that such membrane blebs provide a new, flexible compartment for HAMLET to access critical cellular constituents, notably several activated Ras family proteins on the cytoplasmic face of the plasma membrane and inhibit their downstream activity. Finally, we show that these responses are absent in healthy primary cells, which resist the tumoricidal effects of HAMLET. Membrane integration and perturbation thus provides HAMLET direct access to mechanisms that initiate and drive intracellular processes crucial for survival.

## Results

### HAMLET induces massive tubulation of lipid bilayers

To address if the biological response to HAMLET involves physical membrane remodeling, we first employed protein- and cytosol-free giant unilamellar vesicles (GUVs). The use of GUVs, tens of micrometers in diameter enables real-time visualization of HAMLET-induced dynamics of membrane perturbations using fluorescence microscopy. For visualization, synthetic GUVs^25^ consisting of a single phospholipid (POPC, 1-palmitoyl 2-oleoyl-*sn*-1-glycero-3-phosphocholine) are doped with 1.0–2.0 mol% of a phase sensitive probe (1,2-dioleoyl-sn-glycero-3-phosphoethanolamine-N-(lissamine rhodamine B sulfonyl) (Rho-DOPE)^26^. Suspensions of GUVs containing 300 mM sucrose in an osmotically balanced glucose bath were produced by established electroformation methods^27^.

**Figure 1:**
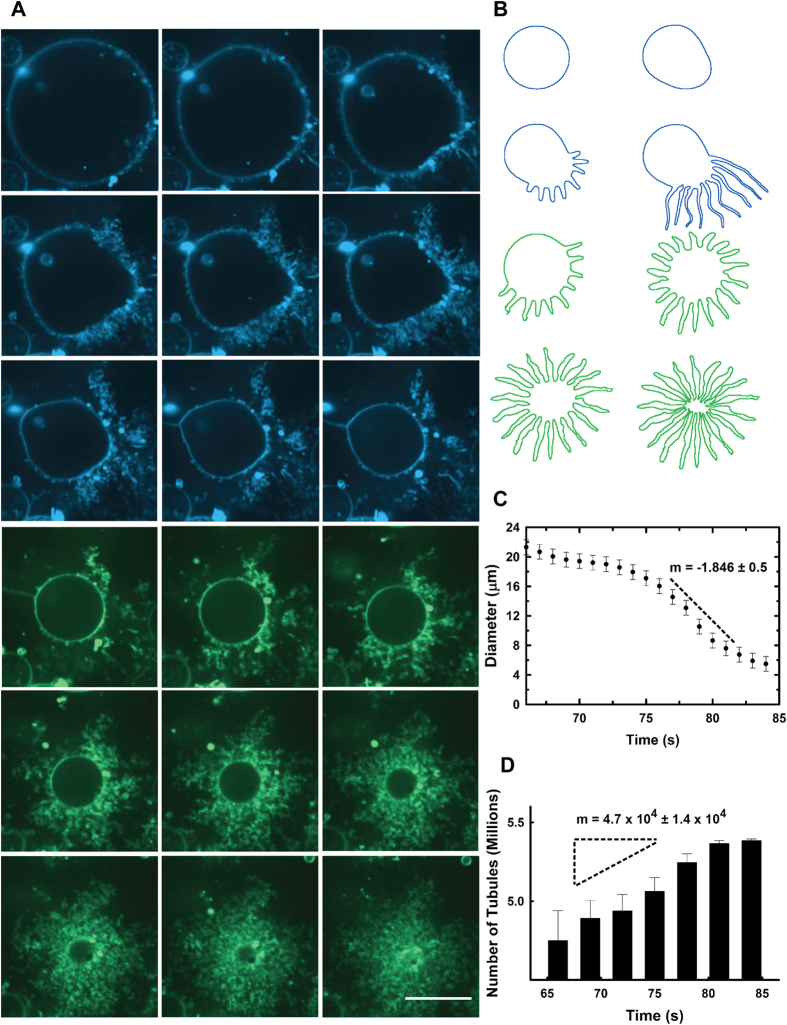
HAMLET induces GUV tubulation. (**A**) Selected equatorial stills from time lapse spinning disk confocal fluorescence images [see Supplementary Movie S1] obtained after incubating POPC GUVs in 30 μM HAMLET. Panels illustrate key steps during the transformation of spherical vesicles into bundles of fluctuating tubules (21 seconds). Fluorescent intensities depicted in false color. Scale bar, 10 μm. (**B**) Cartoon depicting the two transformation sequences in (**A**). (**C**) Plot of the change in GUV diameter over time showing two regimes of shrinkage (rate = −1.846 ± 0.5 μm/s). Systematic errors in measurements of GUV properties are shown. Estimates for diameters are approximate because of deviations from spherical shapes. (**D**) Kinetics of tubule formation showing growth in the number of tubules over time as GUV shrinkage occurs (see methods for calculation). For tubulation kinetics in a population of vesicles, see Supplementary Movie S1.

Using spinning disc confocal fluorescence microscopy, we found that administering HAMLET (30–200 μM) to a population of GUVs prompted a highly dynamic gross membrane remodeling (Supplementary Movie S1A-C). Within seconds, the vesicle assumed a tense spherical shape and within the first hundreds of seconds – some vesicles appeared tubulated, others intact, and still others, with internal membranes (Fig. 1A–D). One of the most striking and frequent changes involved vesicles producing a bundle of fluctuating tubules. Immediately after the addition of HAMLET, the tense spherical shape of the vesicle was replaced by an oblate structure, likely reflecting the initial integration of HAMLET into the membrane (Fig. 1A,B). Concomitantly, membrane tubules began to emanate from the region of the GUV with the highest curvature. *Subsequently*, the lumen of the vesicle shrank and tubules grew up to several micrometers in length, until the GUV was transformed into a rapidly fluctuating bundle of tubes (Fig. 1C,D). The tubules in the bundle were several micrometers long, had diameters near the optical resolution, and exhibited uniform fluorescence intensity consistent with a defined thickness of the tubules. The time scale of the tubulation ranged from several seconds to several tens of minutes depending on the concentrations of HAMLET administered. Even in single experiments, the process was asynchronous and stochastic with some GUVs transforming into tubules within seconds and others more slowly (minutes). Ultimately, all GUVs transformed into tubes, however.

### Membrane integration of HAMLET in GUVs

**Figure 2:**
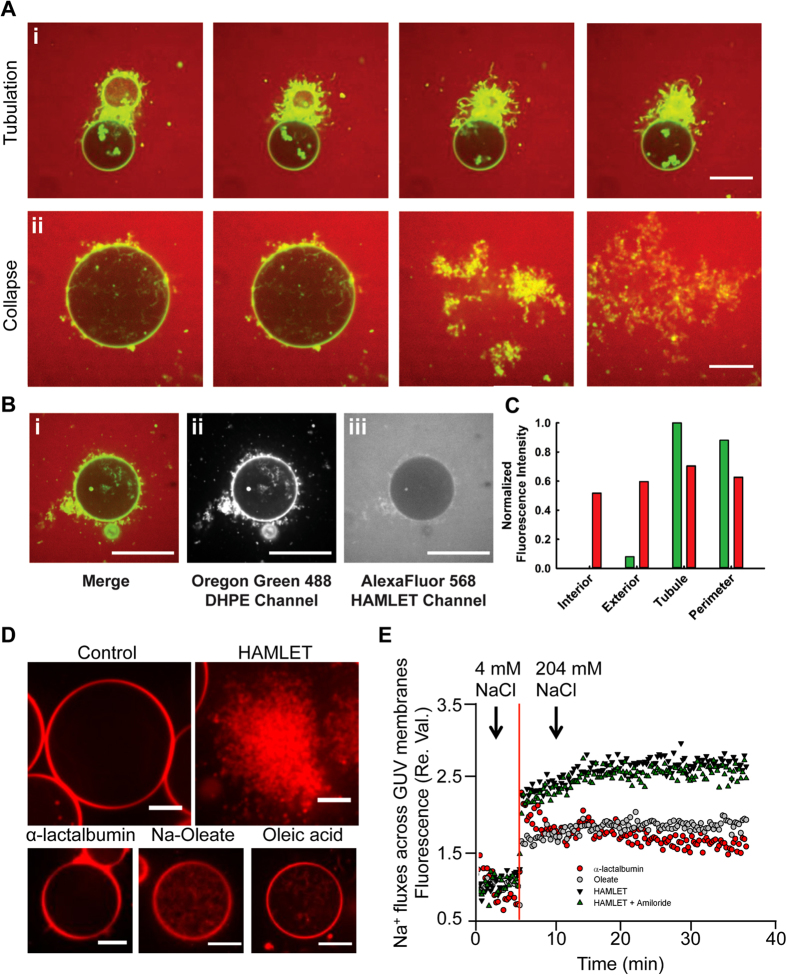
HAMLET interacts with GUVs and triggers ion fluxes across the membrane. (**A**) Selected equatorial stills from a time lapse spinning disk confocal image sequence of a POPC GUV (Oregon Green 488) exposed to 30 μM Alexa-HAMLET [see Supplementary Movies S5, S6 and S7]. Scale bar, 20 μm. (**B**) (i) Merge (ii) Lipid labeled Oregon Green 488 DHPE and (iii) Alexa Fluor 568 colocalize within the tubular deformations of the GUVs. Scale Bar, 60 μm. (**C**) Fluorescence intensity of (b) sampled on the tubule, the perimeter of the GUV, the exterior & interior solutions. Normalized fluorescence intensity was calculated dividing each number by the highest fluorescence value. (**D**) Confocal microscopy images of the incubation of model membrane GUVs with HAMLET individual component compared to untreated control. Scale bar, 10 μm. (**E**) HAMLET (35 μM) induces sodium influx in artificial liposomes compare to HLA (35 μM) and oleate (175 μM) that showed no significant influx.

To determine the fate of the HLA protein in HAMLET, we employed fluorescently labeled, AlexaFluor®568 HAMLET (Alexa-HAMLET), in which the fluorophore was conjugated to HLA via primary amine side chains. GUV experiments, performed using a stained lipid phase (Oregon Green 488 DHPE), revealed that the majority of Alexa-HAMLET, and hence HLA, (>90%) accumulates at the periphery of the membrane tubules (Fig. 2A–C and Supplementary Movies S5–S7). These results confirm that the protein in HAMLET does not partition into any pre-existing membrane evaginations, but rather it is directly responsible for inducing curvature-generating membrane deformations.

To address whether the spherical-to-tubular membrane morphological transition was unique to HAMLET, we carried out experiments in which the individual HAMLET components HLA, oleic acid and sodium oleate were administered at comparable concentrations (Fig. 2D). In the case of HLA, no detectable morphological change was evident (Supplementary Movie S2). Oleic acid and oleate did not induce external tubulation in the GUVs. However, qualitatively different, internal tubules or invaginations were observed (Supplementary Movies S3-S4), comparable to those reported previously^28^,^29^. These results confirm the need for both protein and lipid for HAMLET to produce the external tubulation response in the GUVs.

### HAMLET induces membrane permeabilization in GUVs

To address if model membranes are permeabilized by HAMLET, we quantified ion fluxes across the membranes of small artificial liposomes, exposed to a change from 4mM to 204 mM of NaCl in extra-vesicular concentration, Sodium Green fluorescence was used as readout for the influx of externally added sodium ions (Fig. 2E). Artificial liposomes were produced by mixing 1,2-dioleoyl-***sn***-glycero-3-phospho-(1′-*rac*-glycerol) (DOPG) and Egg yolk L-α-phosphatidylcholine (EYPC) in a 1:1 ratio. HAMLET triggered a rapid influx of Na^+^ ion into the liposomes but neither HLA nor oleic acid had this effect at concentrations equimolar to HAMLET.

To further characterize the effects of HAMLET on membrane integrity, GUVs were incubated with fluorescent, NBD-glucose (58 μM), which does not permeate unperturbed GUVs. After the addition of HAMLET (30 μM), the onset of tubulation did not coincide with the translocation of NBD-glucose into the interior of the tubulating vesicle, suggesting that tubulation per se did not lead to global destabilization of the membrane. It was only after the collapse of the entire vesicular motif, that we witnessed loss of compartmental integrity as seen by the leakage and homogenization of NBD labeled glucose (Supplementary Figure S1A,B). The results suggest that the membrane leakage profile is specific to HAMLET and not reproduced by its subcomponents, HLA and oleic acid.

### Membrane perturbations in tumor cells exposed to HAMLET

**Figure 3:**
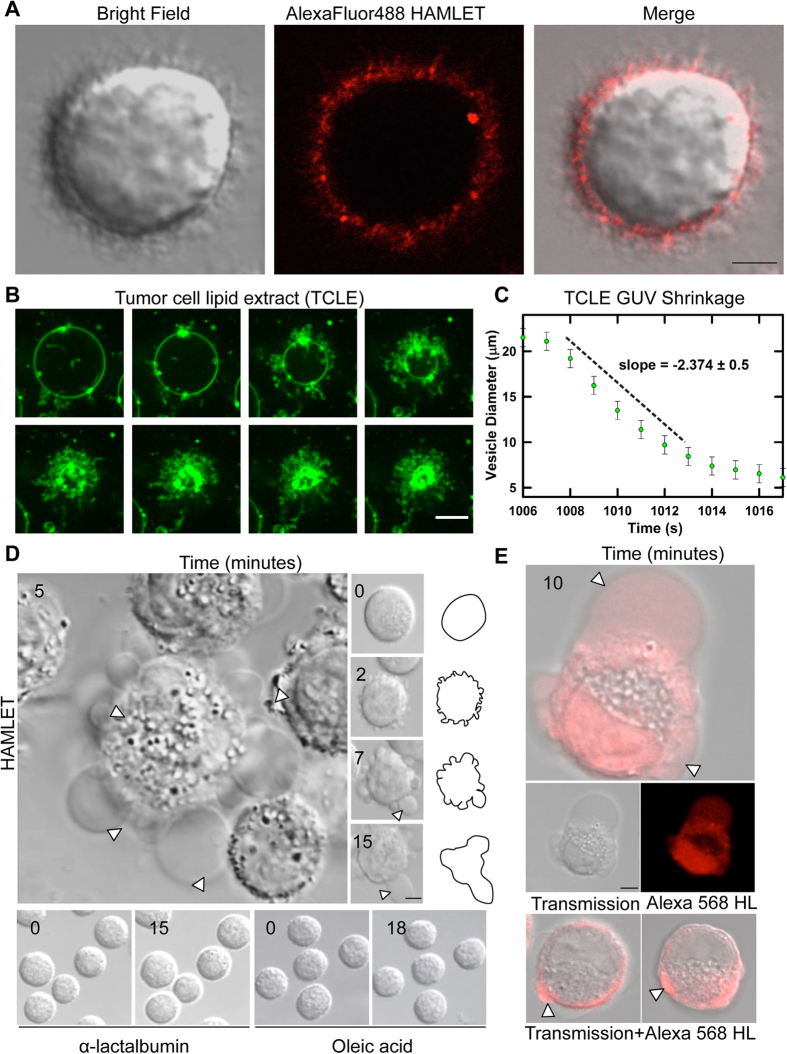
HAMLET interacts with tumor cell membrane (A) Alexa-HAMLET accumulates at the cell membrane at 4 °C with clear evidence of tubulation. Scale Bar, 5 μm (**B**) Fluorescence microscopy images of GUVs formed from A549 lung carcinoma lipid extract (see methods for details) incubated with 30 μM HAMLET (2 seconds between frames). Scale Bar, 10 μm. (**C**) Plot of diameter of vesicle lumen over time, which begins to shrink approximately 16 mins after addition of HAMLET to the GUV exterior. Systematic errors in measurements of GUV properties are shown. Estimates for diameters are approximate because of deviations from spherical shapes. (**D**) Kinetics of membrane protrusion formation in lung carcinoma cell exposed to HAMLET (35 μM). Scale Bar, 5 μm Right panel: Cartoon diagram of (**D**). Bottom panel: shows no significant change in tumor cell morphology in cells treated with HLA (35 μM) and oleic acid (175 μM) alone. (**E**) Alexa-HAMLET accumulates in tumor cell blebs. Scale Bar, 10 μm.

Unlike GUVs, the organization and behavior of lipids in cytoplasmic membranes is constrained by interactions with other constituents, such as membrane proteins and the cytoskeleton^5^. To address if the tubulation response observed in the GUVs has a biological correlate, lung carcinoma cells in suspension were exposed to Alexa-HAMLET and followed by live cell imaging for 5–10 minutes. Rapid membrane tubulation was detected around the cell periphery (Supplementary Figure S2A,B). The tubules showed positive Alexa-HAMLET staining, compatible with membrane integration of HAMLET into the newly formed tubules (Fig. 3A).

The involvement of lipids in the response of tumor cell membranes was addressed, using GUVs made from tumor cell lipid extracts (TCLE). The TCLEs formed GUVs with diameters of 10–30 μm, comparable to those obtained using single purified POPC. The TCLEs formed tubules with kinetics similar to the POPC GUVs, resulting in the shrinkage of the vesicular lumen (Fig. 3B,C and Supplementary Movie S8). The first tubules appeared to emanate from areas of lipid accumulation in the GUV membranes. The results suggest that despite the more complex lipid composition, HAMLET triggers similar membrane responses also in tumor cells.

In addition to tubulation, HAMLET induced gross membrane deformations in A549 lung carcinoma cells, characterized by the appearance of small circular membrane blebs at the cellular periphery, which increased in number during the first 2 minutes. Over the next 5 minutes, the blebs merged, successively increasing in size. After 7 minutes, >80% of tumor cells were affected (Fig. 3D, Supplementary Figure S3A–D and Supplementary Movie S9). The bleb compartments were either polarized or arose around the perimeter of the cells, similar to the deformations found in the GUV model membranes (Fig. 1). Control experiments confirmed that no such blebbing occurred when HAMLET was substituted for the native protein or oleic acid alone (Fig. 3D).

### Membrane blebs creates a new compartment for the interaction of HAMLET with cellular targets

The change in cell morphology suggested that membrane blebs might create a new functional compartment for extracellular ligands like HAMLET to interact with specific cellular targets (Fig. 3E). This hypothesis was addressed by confocal imaging experiments and Ras GTPases were selected as model targets for HAMLET in this analysis. We have recently shown that HAMLET specifically recognizes this family of GTPases and perturbs their function^30^. Of 16 Ras family proteins that were targeted by HAMLET *in vitro*, 14 (87.5%) can be stably membrane anchored because of post-translational lipidation of cysteine residues at the *CAAX* motif^31^.

**Figure 4:**
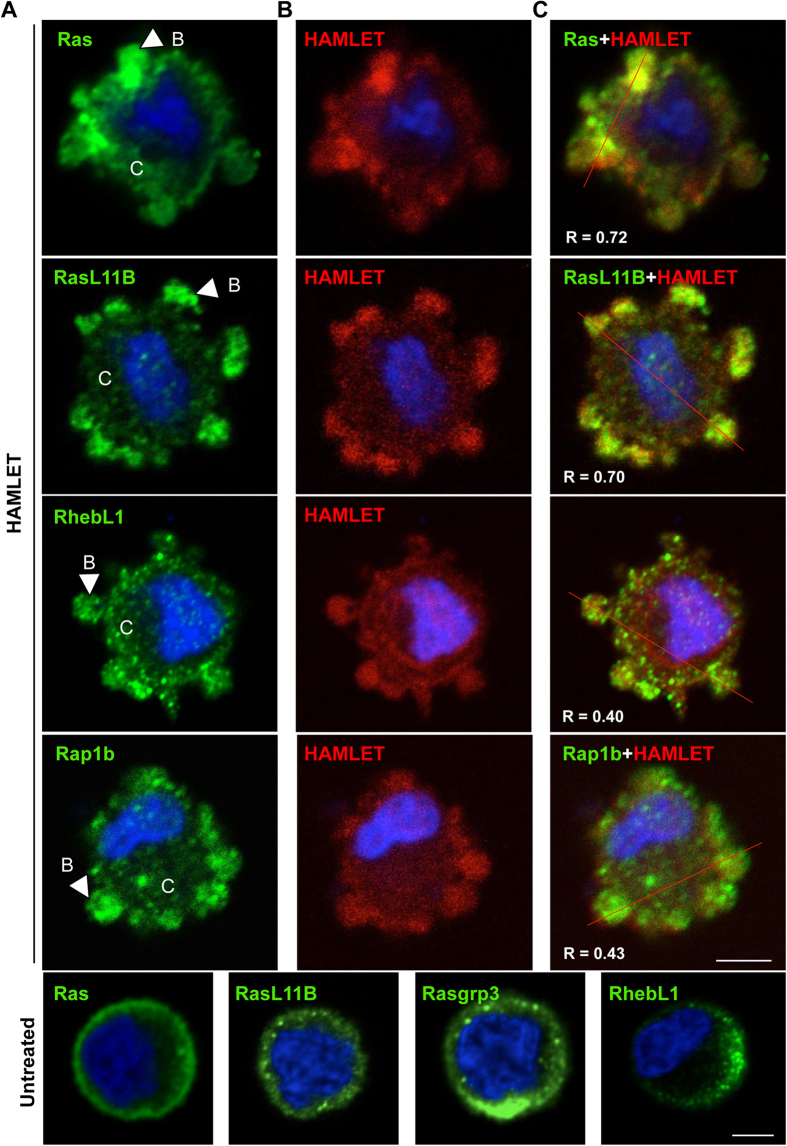
Accumulation of Ras family proteins in membrane protrusions. (**A**) Ras, RasL11B, RhebL1 and Rap1B (green) accumulate in membrane blebs, 15 minutes after HAMLET challenge (35 μM). Arrows indicates blebbing structures, B = blebs, C = Cytoplasm. (**B**) Alexa-HAMLET (red) shows a similar pattern of membrane localization. (**C**) Merged confocal images. Untreated cells are shown in the bottom panel. R = Pearson coefficient for co-localization. Scale bar, 5 μm.

To address if the integration of HAMLET into the membrane blebs might facilitate interactions with activated Ras, lung carcinoma cells were exposed to Alexa-HAMLET (35 μM) and the cells were counterstained for the 16 Ras family proteins. Rapid accumulation of Ras proteins in membrane blebs was detected after 15 minutes (Fig. 4 and Supplementary Figure S4,S5). RasL11B, a recently discovered Ras family member, whose function has not been characterized, showed a clear redistribution to membrane blebs around the cellular periphery, as did RhebL1, which regulates mTOR signaling, and Rap1B, which is involved in proliferation. Ras was clustered in a few larger membrane protrusions. Braf also showed rapid accumulation in the membrane blebs, as did ArfGAP3, Rab3C and Arl5A. Raf1, which is a Braf homologue controlling signaling downstream of Ras and the Ras activator Rasgrp3, showed staining both at the membrane and throughout the cells (Supplementary Figure S4–S6).

The results suggest that membrane blebs formed in response to HAMLET, provide a new compartment, spatially confining HAMLET in the vicinity of critical signaling clusters, exemplified by Ras family proteins.

### Lack of membrane tubulation, blebbing or Ras accumulation in healthy, differentiated cells

**Figure 5:**
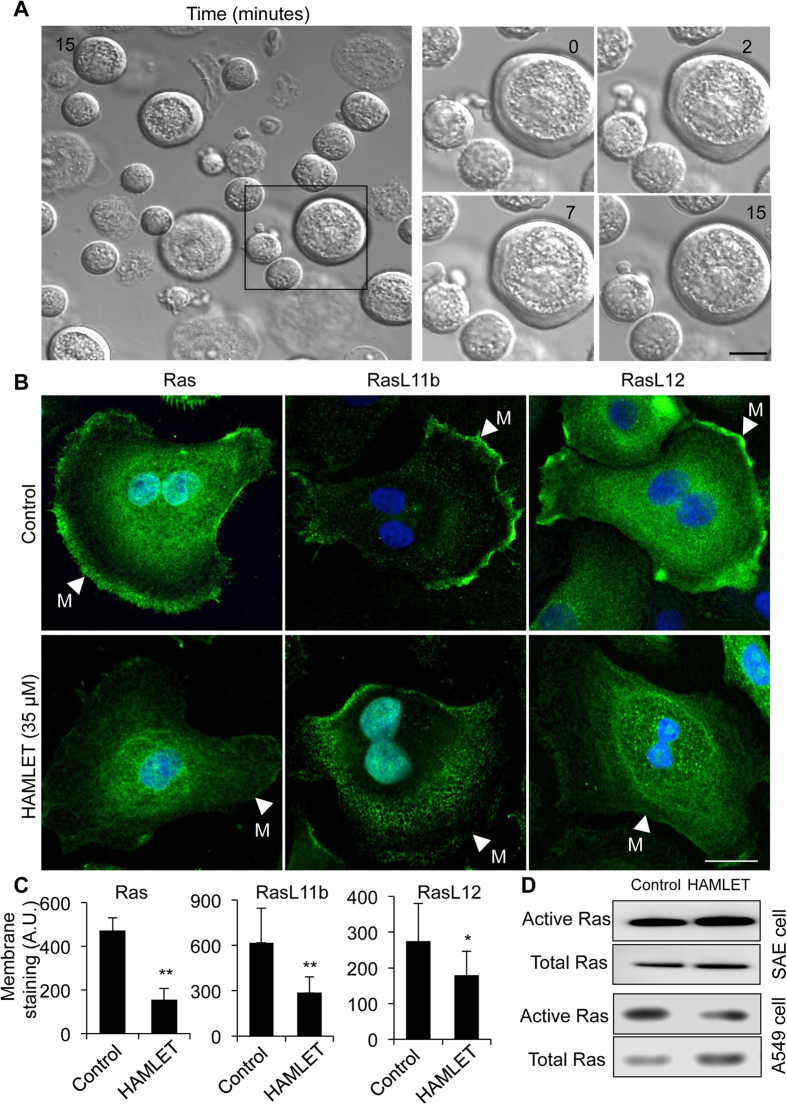
Insensitivity of healthy, differentiated cell membranes to HAMLET challenge. (**A**) Maintained membrane integrity and structure in healthy small airway epithelial cells (SAEs) after HAMLET exposure (35 μM, 15 minutes). Scale Bar, 5 μm (**B**) Loss of Ras proteins from the membrane (arrows). Fixed cells stained for Ras, RasL11B or RasL12. Scale bar, 10 μm. (**C**) Quantification of Ras, RasL11B or RasL12 staining in SAE cells treated with HAMLET or untreated control (n = 10). (**D**) Quantification of Ras activity in control untreated and HAMLET-treated SAE and A549 cells. The active Ras was pulled down with the Ras-binding domain of Raf1.

Healthy, differentiated cells are more resistant to HAMLET than lung carcinoma cells and maintain their viability. If membrane perturbations characterize the tumor cell response to HAMLET, we would not expect membrane changes to occur in healthy primary cells. To address this question, small airway lung epithelial cells, in primary culture, were exposed to HAMLET and the membrane response was monitored by real-time confocal microscopy, for 20 minutes (Fig. 5A). Membrane tubulation was not detected and membrane blebs were not formed, in contrast to the tumor cells, where membrane changes occurred almost instantaneously. HAMLET bound to the membranes of the SAE cells without evidence of tubulation (Supplementary Figure S7), indicating that the membrane perturbation is the decisive factor and not HAMLET recognition *per se.* Furthermore, the redistribution of Ras proteins differed markedly from that in tumor cells. Rather than membrane accumulation, Ras, RasL11B and RasL12 were redistributed from the membrane of untreated SAE cells to intracellular compartments (Fig. 5B,C and Supplementary Figure S8,S9). Inhibition of Ras activity by HAMLET in lung carcinoma cells was demonstrated by co-immunoprecipitation of active Ras with the Ras-binding domain of Raf1. In contrast, no effect of HAMLET on Ras activity was recorded in healthy, differentiated cells (Fig. 5D).

As the Ras GTPases are critical for cell proliferation and survival, these observations support the notion that membrane responses to HAMLET may influence the difference in susceptibility and cell death.

## Discussion

HAMLET is a protein-lipid complex with broad tumoricidal activity and therapeutic efficacy in tumor models and clinical studies. The broad-spectrum efficacy of HAMLET in killing vastly different tumor cells suggests that HAMLET must target molecules that are prevalent in tumor cells and that control conserved cell-death pathways. To this end, a discrete and susceptible target is the lipid bilayer component of the tumor cell membrane. But how HAMLET binds the exoplasmic leaflet of the cell membrane and how it affects the cellular membrane has largely been unknown. Previous studies purportedly examining HAMLET-membrane interactions suggest pore formation in model membrane monolayers^32^, as well as morphological distortions, loss of structural integrity, and permeabilization in bilayers and tumor cells^21^,^33^. In the absence of real-time dynamic measurements, these observations do not lend themselves to a unifying view on the types (and mechanisms) of membrane morphological remodeling induced by HAMLET.

The appearance of tubules in giant vesicles devoid of proteins or energy generating machinery suggests that the insertion of HAMLET itself is sufficient to trigger tubulation. Unlike surface docking of proteins at high concentrations that generate crowding in domain-forming membranes or intracellular and endogenous scaffolding proteins such as COPII or BAR proteins, which act both as sensors and inducers of membrane curvature^8^,^13^, HAMLET is a more complex exogenous curvature generator, acting through membrane insertion from the extracellular space. We propose that the appearance of tubular extensions is a direct consequence of the insertion of both the partially unfolded protein and multiple, amphiphilic oleic acid ligands into the membrane, which creates conditions for the generation of local spontaneous curvature, as described previously for multi-anchoring polymer^34^. It thus appears that the lipid membrane itself can provide the cell with mechanisms for sensing external ligands and translating physical processes into the language of signal transduction.

Assuming HLA to oleic acid stochiometry to be between 1: 4 and 1: 6 in HAMLET, we estimated the concentrations of pure components that mimic those delivered by HAMLET to be 175 μM for oleate and 30 μM for HLA for 30 μM HAMLET concentration. Conducting experiments using these concentrations of oleic acid (175 μM), sodium oleate (175 μM), and HLA (30 μM), we find that individual components do not reproduce the deformations (i.e., generation of tubular evaginated protrusions); even concentrations as high as 500 μM failed to produce comparable tubules. Specifically, HLA fails to produce any observable deformations and OA and sodium oleate induces internal (rather than external) tubulation – albeit to a limited and variable extent. We reason that this dramatic disparity in the effects of HAMLET and pure components might originate from the differences in the manners by which oleate component interacts with membrane bilayers. HAMLET locally inserts multiple amphiphiles in the membrane driving spontaneous curvature generation needed to drive protrusion formation such as we observe. We draw parallel with a qualitatively similar membrane tubulation reported for synthetic polymers presenting multiple amphiphilic side-chains reported previously^35^.

Reconciling these observations with models for interactions of oleic acid with phospholipid membranes^36^,^37^, we deduce that the HAMLET-membrane interactions occur through a novel mechanism, distinct from those characterizing membrane remodeling by typical curvature-inducing scaffolding proteins (e.g., BAR domains and COPII)^8^,^13^, due to (1) local delivery and insertion of multiple, protein-bound oleate cofactors to the exoplasmic membrane leaflet and (2) a reduction in diffusive flip-flop (or translocation) of the oleic acid between the bilayer leaflets. A net synergistic effect of the interplay of these two factors is to stabilize membrane asymmetry, driving the creation of a net area-difference between the membrane leaflets and generating an effective spontaneous curvature. These alterations in the membrane compositions and geometry then give rise to membrane tubulation, such as we observe. Since the physical principles underlying this membrane interaction and uptake mechanisms are not specific to HLA alone, and may be recapitulated by LA species variants (e.g., bovine, equine, and caprine lactalbumins), these results might provide a mechanistic basis for appreciating the diversity of proteolipid complexes, which show potential for tumoricidal activity^38^.

HAMLET’s interaction is driven by mechanisms distinctly different from those produced by protein crowding at the membrane surface. Unlike surface docking of proteins at high concentrations that generate crowding in domain-forming membranes, HAMLET is itself a meta-amphiphile consisting of unfolded α-lactalbumin and a discrete number of oleic acid residues. Importantly, the protein component of HAMLET, namely, human a-lactalbumin, retains membrane-binding capacity but does not induce tubulation. Similarly, oleic acid alone is also incapable of generating comparable outwardly directed tubulation at the concentrations used (although some internal tubulation is evident). These lines of evidence then emphasize the critical role played by the unique, complex geometry of HAMLET. We have reasoned that the oleic acid cofactor, which naturally partitions within the hydrophobic core of the membrane, produces area difference between the two leaflets; a reduction in diffusive flip-flop (or translocation) of the oleic acid between the bilayer leaflets due to HLA-oleate association in HAMLET then drive tubule generation by well-known curvature generation mechanisms.

Membrane blebbing has been investigated as a mechanism involved in ameboid cancer cell motility, promoting the invasiveness of metastatic cells^39^. Rho and Roc dependent blebbing can be triggered in normal cells by the loss of p53^40^. In addition, in tumor cells, Src kinases activate blebbing and motility through the SH4-domain, which facilitates tumor cell invasion^41^,^42^,^43^,^44^,^45^,^46^. While motility has not been addressed, cell death in response to HAMLET is independent of p53^47^. In particular, canonical apoptotic regulators such as caspases and Bcl-2 have also been ruled out, based on Bcl-2 overexpression and caspase inhibition experiments. Furthermore, in preliminary studies, the ROC kinase has not been found not to influence HAMLET-induced cell death. Importantly, this suggests that HAMLET circumvents fundamental anti-apoptotic strategies adopted by tumor cells.

These findings illustrate a more general physical mechanism for receptor-free signaling, where the docking and insertion of extra-cellular protein-lipid complexes may produce membrane conformations, which serve as surrogate signaling receptors. It appears tempting to speculate that the ability of HAMLET to induce and select membrane conformations to localize and initiate signaling may illustrate a more pervasive class of physical mechanisms for ‘’receptor-free” signaling – presumably prevalent in early protocells – where docking, crowding, and insertion of extra-cellular proteins modulate membrane conformations. The conceptual departure from the receptor-dependent lock-and-key model of signal transduction, exemplified here, does not rule out the importance of specificity, but it causes us to re-evaluate the notion of a specific receptor as a prerequisite for cellular recognition.

## Materials and Methods

### Materials

1-palmitoyl-2-oleoyl-sn-glycero-3-phosphocholine (POPC) and Rhodamine-B DOPE (lissamine rhodamine B 1,2-dioleyl-sn-glycero-3-phosphoethanolamine) were from Avanti Polar Lipids (Birmingham, AL), Oregon Green 488 1,2-dihexadecanoyl-sn-glycero-3 phosphoethanolamine (Oregon Green DHPE) from Life Technologies (Carlsbad, CA), Oleic acid, sodium oleate, and glucose from Sigma-Aldrich (Saint Louis, MO) and sucrose from EMD Chemicals (Philadelphia, PA). Rabbit anti-Ras (EPR3255), Rasgrp3 (AB103622), Rab3c (AB170053), and mouse anti-RasL12 (AB67622) and Rhebl1 (AB57333) were from Abcam (Cambridge, Cambridgeshire, UK). Rabbit anti-Rap1A/B (#4938S), rabbit anti-Raf1 (C-12), Arl5A/5C/8 (H-113), were from Cell Signaling (Danvers, MA) and mouse anti Raf-B (F-7), RasL11b (5J7) and Arfgap 1/3 (G-11) from Santa Cruz (Dallas, TX). RPMI-1640 was from HYclone (HYCLSH30027); sodium pyruvate (11481318), non-essential amino acids (11401378) and fetal bovine serum (10309433) were from Fisher Scientific; gentamicin was from Life Technologies (15710049); A549 cells (ccl-185), primary small airway epithelial cells (PCS-301-010), airway epithelial cell basal medium (PCS-300-030) and small airway epithelial cell growth kit (PCS-301-040) were from ATCC.

### Giant unilamellar vesicle experiments

Giant vesicles were prepared by adapting the well-established electroformation method due to Angelova and co-workers^27^. Small droplets (15–25 μL) of lipid solution in chloroform (2 mg/ml) were spread on an ITO substrate and allowed to dry under vacuum for 1 h. The dried lipid cake was then hydrated with a 300 mM sucrose solution in deionized water and sandwiched using a second ITO slide. GUVs were electroformed by subjecting the sandwich to A 3V AC sine-wave voltage at 10 Hz for 2 h followed by a 3V square wave voltage at 2 Hz for an additional 1 h. For study, GUV solution was diluted to a final lipid concentration of 0.01 mg/ml.

HAMLET, HLA, oleic acid, and sodium oleate were incubated at room temperature with the diluted GUV suspension. GUVs were incubated with 30 μM (HAMLET and HLA) or 175 μM (oleic acid and sodium oleate) solution for a period of 30 min to 2 h and imaged under a fluorescence microscope in spinning disk confocal configuration.

### Preparation of Tumor cell lipid extract (TCLE) giant unilamellar vesicle

A549 lung carcinoma cell lipids were extracted into chloroform/methanol (2:1) using the Folch Method^48^. From the lipid extract a stock solution was prepared consisting of TLCE/Rhodamine-B DOPE (98/2 by mass). Lipids were subsequently dried on the conductive sides of ITO slides as described above. In a modification of the above procedure, the lipids were then prehydrated in an oven at ~50 °C containing saturated K_2_SO_4_ solution for ~24 hr. The slides were then assembled for electroformation with 300 mM sucrose as used above. The electroformation parameters were a 500 hz sine wave for 3 hours.

### Flux and leakage assay

Flux assays in artificial liposomes were performed using Sodium Green (Invitrogen) fluorescence. Artificial liposomes were produced by mixing chloroform solubilized 1,2-dioleoyl-***sn***-glycero-3-phospho-(1′-*rac*-glycerol) (DOPG, Avanti Lipids) and Egg yolk L-α-phosphatidylcholine (EYPG, Avanti Lipids) in a 1:1 ratio. Chloroform was removed by evaporation in a dry nitrogen stream and lipids where washed in anhydrous pentane. Pentane was evaporated in a dry nitrogen stream and the lipids where solubilized in buffer 1 (20 mM TRIS, 4 mM NaCl, 34 mM CHAPS, 5 μM Sodium Green, pH 7.5) via bath sonication. Following 1 h incubation, CHAPS was removed by centrifuge assisted gelfiltration: Sephadex G50 (Sigma-Aldrich) where swollen in Buffer 2 (20 mM TRIS, 4 mM NaCl, 5 μM Sodium Green, pH 7.5) and packed in Pierce Disposable Plastic columns. Lipid solution was loaded and buffer-shift was accomplished by centrifugation at 1000 × g for 1 min. Liposomes where prepared by seven freeze-thaw cycles using liquid N_2_ and a 55 °C water bath. Liposomes were exposed to 10 s sonication before Sodium Green was removed from the extraliposomal environment by centrifuge-assisted gelfiltration in buffer 3 (20 mM TRIS, 4 mM NaCl, pH 7.5). Liposomes where subsequently loaded into microwell plates and incubated with HAMLET, α-lactalbumin or oleate before flux measurements performed using a Fluostar-Optima, BMG microplate reader. Sodium Green was excited at 485 nm and fluorescence was continuously measured above 520 nm during a 5 min stabilization period, after which 200 mM NaCl was automatically added to induce Na^+^ influx (2 μl buffer 4 (20 mM TRIS, 2 M NaCl, pH 7.5) to 200 μl).

Leakage of GUVs consisting of POPC was further investigated with fluorophore labeled 2-NBDG; GUVs were electroformed in 300 mM sucrose. Prior to incubation with 30 uM HAMLET, vesicles were then diluted in PBS containing 58 μM 2-NBDG in the GUV exterior to monitor leakage.

### Spinning disk confocal fluorescence microscopy

Experiments were performed using Intelligent Imaging Innovations Maranias Digital Microscopy Workstation (3i Denver, CO) fitted with with a CSU-X1 spinning disk head (Yokogawa Musashino-sh, Tokyo, Japan), an Quantem512SC EMCCD camera (Photometrics Tuscon, AZ), and multi-laser stack (50 mW, for illumination. Images were obtained using oil immersion objectives (Zeiss Fluor 40x (NA 1.3) and a Zeiss Plan-Fluor 63x (NA 1.4), Carl Zeiss Oberkochen, Germany) and processed using ImageJ (http://rsbweb.nih.gov/ij/) and Slidebook digital microscopy imaging software (3i Denver, CO).

### HAMLET: characterization, production and quality control

α-Lactalbumin was purified from human breast milk by 25% ammonium sulphate precipitation and hydrophobic interaction chromatography, partially unfolded and converted to HAMLET on a oleic acid-conditioned DEAE ion exchanger^49^. Human milk was obtained from individual donors, after signed informed consent. Each donor was aware that the samples might be used in scientific research. The samples were de-identified and steps were taken to protect the participants’ identities. The procedure was approved by the human ethics committee of the Medical Faculty, Lund University, Lund, Sweden.

### Mammalian Cell Culture

Lung carcinoma cells (A549) and primary small airway epithelial cells (SAE cells) were procured from American Type Culture Collection (ATCC). Lung carcinoma cells were maintained in RPMI-1640 medium supplemented with 1 mM sodium pyruvate (Fisher Scientific), non-essential amino acids (1:100) (Fisher Scientific), 50 μg/ml gentamicin (Gibco, Paisley, UK) and 5% fetal calf serum (FCS). Small airway epithelial cells were maintained in airway epithelial cell basal medium (ATCC) supplemented with small airway epithelial cell growth kit (ATCC), according to ATCC instructions. Cells were cultured at 37 °C, 90% humidity and 5% CO_2_.

### Confocal imaging

Lung carcinoma cells treated with HAMLET (35 μM, 10% Alexa-HAMLET labeled with AlexaFluor568 or AlexaFluor488 via amine coupling according to manufacturer’s instructions (Life Technologies)). Cells were fixed with paraformaldehyde (4%), permeabilized with Triton X (0.25% in PBS) for 10 min and incubated with primary antibodies (1:25 dilution in 10% FCS/PBS) for 2 h at room temperature, washed with PBS (three times) and then incubated with appropriate secondary antibodies conjugated to Alexa-488 (1:100 in 10% FCS/PBS, Molecular Probes). Nucleus was stained with DRAQ-5 (Abcam), cells were washed with PBS three times and mounted on a polylysine slides (Thermoscientific). Slides were examined using LSM 510 META laser scanning confocal microscope (Carl Zeiss). Colocalization analysis and fluorescence quantification were performed in LSM510 image browser software and Photoshop CS5, respectively. For quantification of Ras family proteins in lung carcinoma cells, individual tumor cell bleb (n = 3 to 8/cell) was outlined as region of interest (ROIs) and compared to cytoplasmic staining of the same cell (n = 6). The mean gray scale values were calculated with Photoshop CS5 and expressed as fluorescence intensity. For Ras, RasL11B and RasL12 staining in primary small airway epithelial cells, individual cell membrane regions (n = 10) were outlined as regions of interest (ROIs), and the total fluorescence intensities were measured using LSM 510 image browser software.

Images were captured using LSM 510 META confocal microscope (Carl Zeiss, 40x oil immersion objective, 633 nm HeNe laser and 650 long-pass filter, 488 nm Argon/2 laser and 505–530 band-pass filter, 543 nm HeNe laser and 585–615 band-pass filter).

### Live transmission light microscopy imaging

Membrane changes in lung carcinoma cells or primary small airway epithelial cells were visualized by transmission light microscopy imaging. Cells were seeded on a glass cover slip and allowed to partially adhere to the glass surface for 10 min at room temperature prior to HAMLET treatment (35 μM). Immediate changes in cell morphology were captured with LSM 510 META confocal microscope (Carl Zeiss) using 40× oil immersion objective.

### Membrane localization of HAMLET

Lung carcinoma cells or primary small airway epithelial cells in suspension were treated with HAMLET (35 μM, 10% Alexa-HAMLET) at 4 °C for 30 min, unbound HAMLET was washed with cold PBS and images were acquired with LSM 510 META confocal microscope (Carl Zeiss) using 40x oil immersion objective, 488 nm Argon/2 laser and 505–530 band-pass filter).

### Ras activity measurements

Lung carcinoma cells or primary small airway epithelial cells were treated with HAMLET. Cell lysates were prepared and 1 mg/ml of protein was used for pull down of active Ras complexes with GST tagged RBD. Immunoprecipitation was done according to manufactures instructions (Thermoscientific). Immunoprecipitated samples were separated on Bis-Tris (4–12%) SDS gel (Invitrogen), the separated proteins were blotted to polyvinylidine fluoride (PVDF) membranes (GE Health Care) and probed with mouse anti-Ras antibody (1:200 dilution in 3% BSA, Thermoscientific) for 2 h at room temperature. PVDF membranes were washed with PBS Tween, 0.1% (PBST) and probed with secondary antibody, goat anti-mouse-HRP (1:4000 dilution in 5% milk, DAKO) for 1 h at room temperature followed by washing three times with PBST. Active and total Ras were visualized using ECL chemiluminescence reagent (GE Healthcare).

### Statistical analysis

Pearson product-moment correlation coefficient, R, was performed for co-localization analysis. Student’s T-test was used to calculate statistical significance of the experiments (Microsoft Excel).

## Additional Information

**How to cite this article**: Nadeem, A. *et al.* Protein receptor-independent plasma membrane remodeling by HAMLET: a tumoricidal protein-lipid complex. *Sci. Rep.* **5**, 16432; doi: 10.1038/srep16432 (2015).

## Supplementary Material

Supplementary Information

Supplementary Movie S1A

Supplementary Movie S1B

Supplementary Movie S1C

Supplementary Movie S2

Supplementary Movie S3

Supplementary Movie S4

Supplementary Movie S5

Supplementary Movie S6

Supplementary Movie S7

Supplementary Movie S8

Supplementary Movie S9
