# Supplementary material for: Protein receptor-independent plasma membrane remodeling by HAMLET: a tumoricidal protein-lipid complex
Source: Sci Rep. 2015 Nov 12;5:16432. doi: 10.1038/srep16432 (PMC4642337; doi:10.1038/srep16432)
Supplement: Supplementary Information [file srep16432-s1.doc]

Supplementary Materials for

**Proteins receptor-independent plasma membrane remodeling by HAMLET; a tumoricidal protein-lipid complex**

Aftab Nadeem^1^, Jeremy Sanborn^2^, Douglas L. Gettel^1^, James Ho C.S.^1,3^, Anna Rydström^2^, Viviane N. Ngassam^1^, Thomas Kjær Klausen^4^, Stine Falsig Pedersen^4^, Matti Lam^1^, Atul N. Parikh^2,3^* Catharina Svanborg^2^ *

*^1^Department of Microbiology, Immunology and Glycobiology (MIG), Institute of Laboratory Medicine, Lund University, S-223 62 Lund, Sweden*

^2^*Departments of Applied Science, Biomedical Engineering, and Chemical Engineering & Materials Science, University of California, Davis, CA 95616 USA*

*^3^Centre for Biomimetic Sensor Science, School of Materials Science and Engineering, Nanyang Technological University, 50 Nanyang Drive, 637553, Singapore*

*^4^Department of Biology, University of Copenhagen, Universitetsparken 13, 2100, Copenhagen, Denmark.*

*Correspondence and requests should be addressed to

C. S. ([catharina.svanborg@med.lu.se](mailto:catharina.svanborg@med.lu.se)) or A. N. P. ([anparikh@ucdavis.edu](mailto:anparikh@ucdavis.edu)).

**Supplementary Material and Methods**

Equating the change of volume of the source vesicle with the volume encapsulated by newly formed tubes, a simple relationship between number of tubes and tube diameters with the time-dependent vesicle diameters can be written:


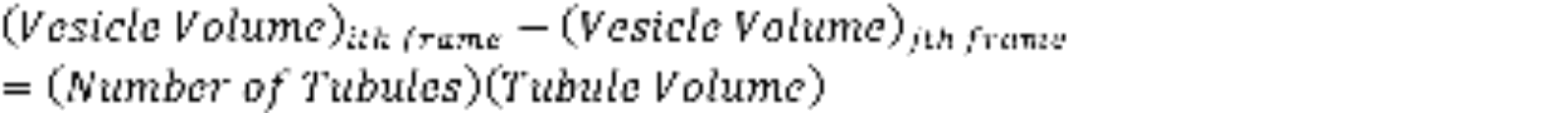


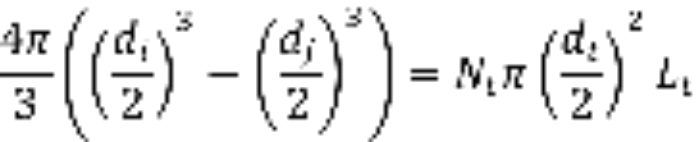


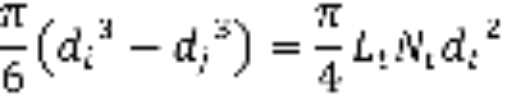


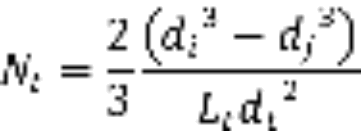


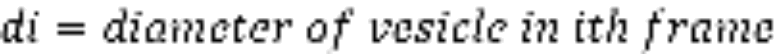


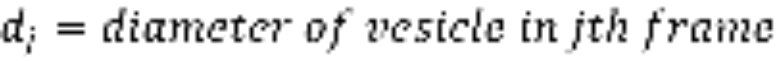


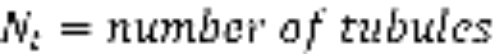


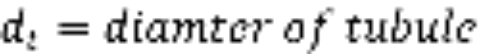
 (assumed to be 0.1 µm)


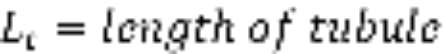
 (assumed to be 1 µm)

This requires the assumptions that vesicles remain spherical and that the encapsulated volume during HAMLET induced tubulation is fixed. Quantifying the tubulation process, even using this simple consideration, is not accurate because of inherent loss of information is two-dimensional confocal fluorescence slices we image. Nonetheless, a rough estimate for the number of tubules formed can be obtained by assuming the average tubule length of 1 um (evident in data) and tubule diameter of 0.1 um from comparable previous data of protein-induced membrane tubulation (Tanaka-Takiguchi, Itoh et al. 2013), an accurate determination of which for the case at hand requires independent electron microscopy measurements (not performed here).

**Supplementary Reference**

Tanaka-Takiguchi, Y., T. Itoh, K. Tsujita, S. Yamada, M. Yanagisawa, K. Fujiwara, A. Yamamoto, M. Ichikawa and K. Takiguchi (2013). "Physicochemical Analysis from Real-Time Imaging of Liposome Tubulation Reveals the Characteristics of Individual F-BAR Domain Proteins." Langmui**r** 29(1): 328-336.

**
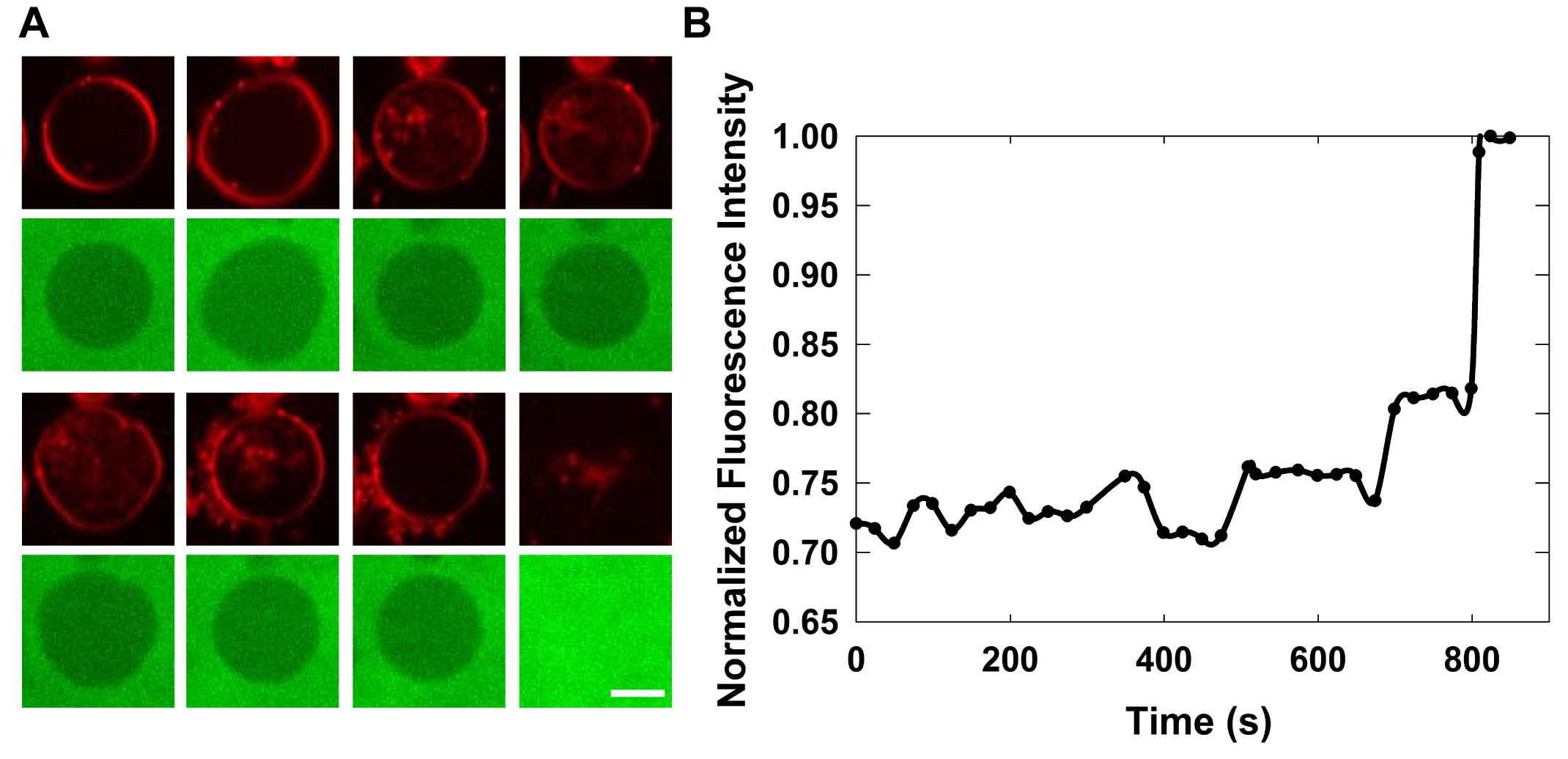
**

**Supplementary Figure S1: Leakage Assay of POPC GUVs incubated with HAMLET.** (**A**) Selected stills from spinning disk confocal fluorescence image sequences of POPC GUVs doped with 1% Rho-B DOPE incubated with 30 μM HAMLET. Leakage is monitored using 58 µM 2-NBDG in the GUV exterior. (**B**) Quantification of fluorescence intensity of intravesicular 2-NBDG at different time points. Fluorescence intensity values were normalized by dividing each individual value with the highest fluorescence value. Scale bar,10 μm.


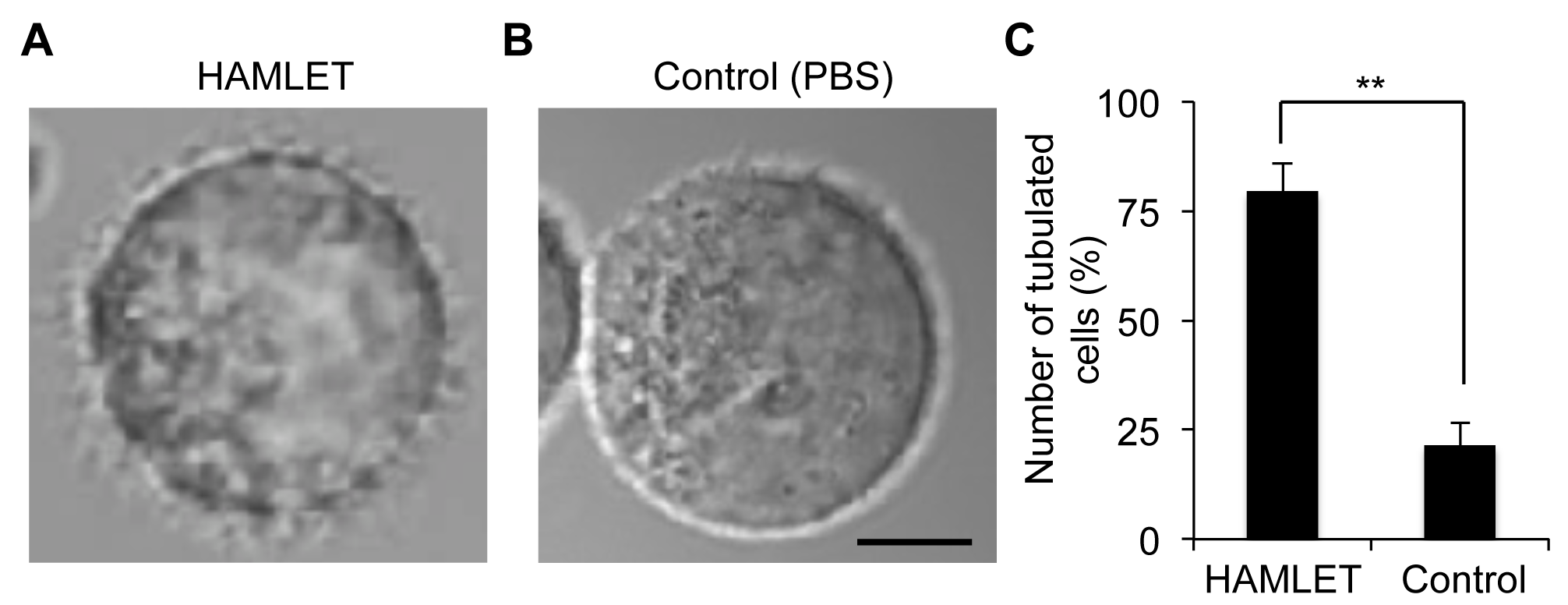


**Supplementary Figure S2: Differences in tubule formation between control (PBS) and HAMLET treated cells. A)** Tubulation in HAMLET treated cells. **B)** Low tubulation in untreated cells (PBS). Scale bar, 5 μm. **C)** Quantification of the difference in tubule formation. **p<0.01

**
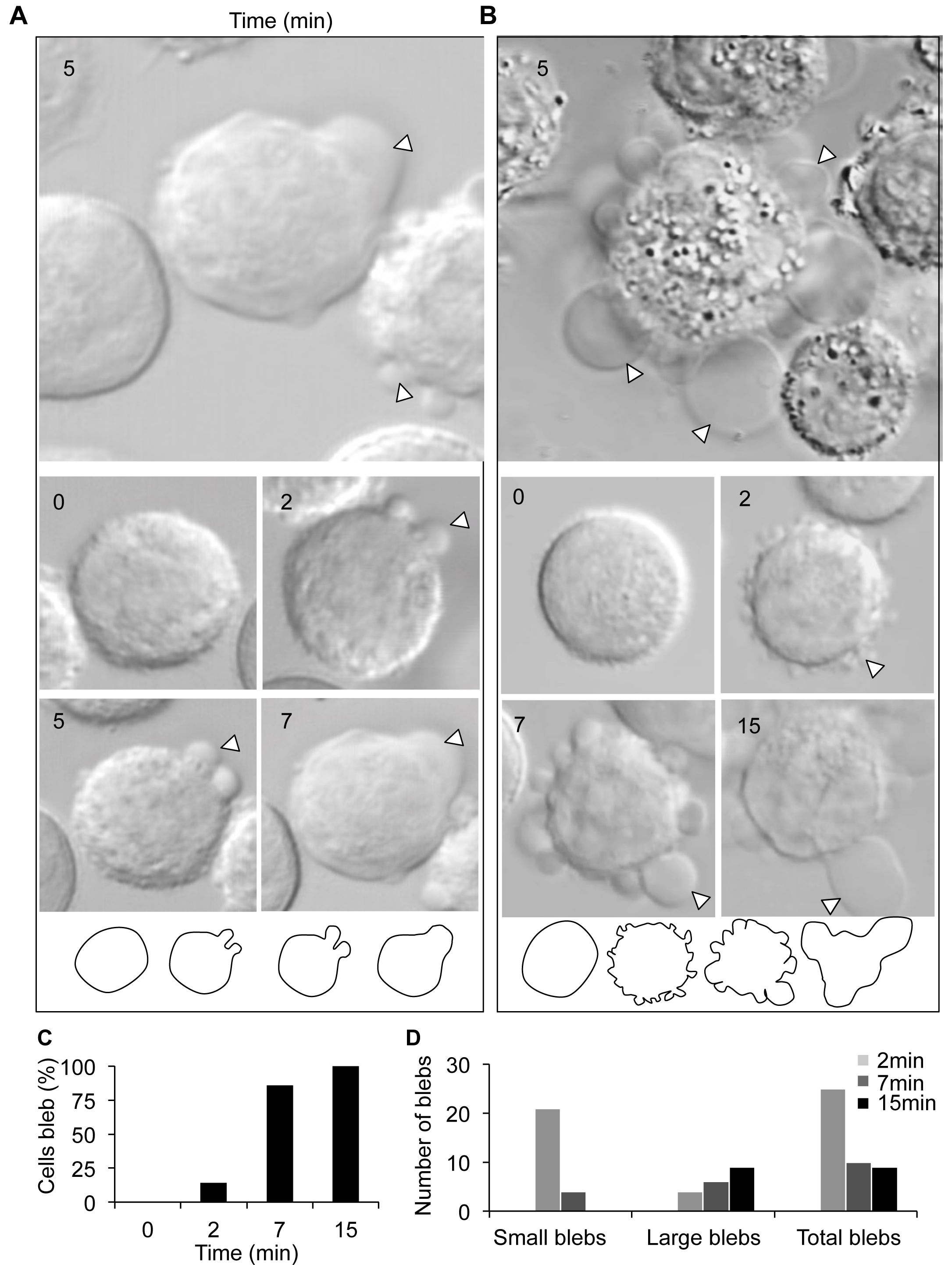
**

**Supplementary Figure S3: HAMLET induced vesicle formation in tumor cell membranes.** Live images were captured by confocal microscopy at different times after HAMLET exposure of lung carcinoma cells. (**A)** Population of cells with polar vesicles **(B)** Population of cells with circumferential vesicles **(C)** Kinetics of HAMLET-induced tumor cell blebbing **(D)** Decrease in the number of small blebs and increase in large blebs over time. Scale bar, 5 μm.

**
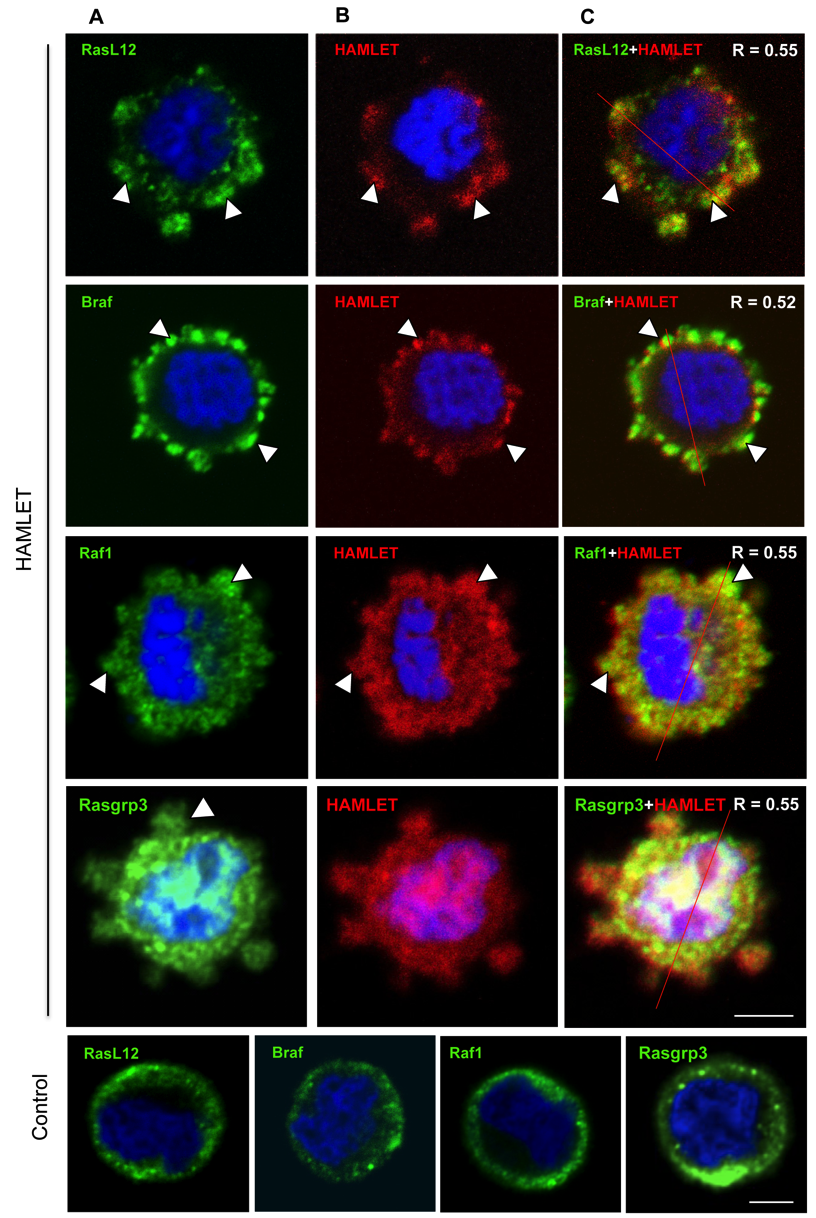
**

**Supplementary Figure S4: Accumulation of Ras family proteins in membrane protrusions. (A)** Confocal images show the enrichment of different Ras proteins (green) (RasL12, Braf and Raf) in tumor cell blebs compared to the cytoplasmic region. Rasgrp3 was present in membrane blebs but was more abundant in the cytoplasm. **(B)** HAMLET (red) was also present in the membrane blebs. **(C)** Merged confocal images. Red lines represent intensity distribution region used for calculating Pearson coefficient. R = Pearson coefficient for co-localization. Scale bar, 5 μm.

**
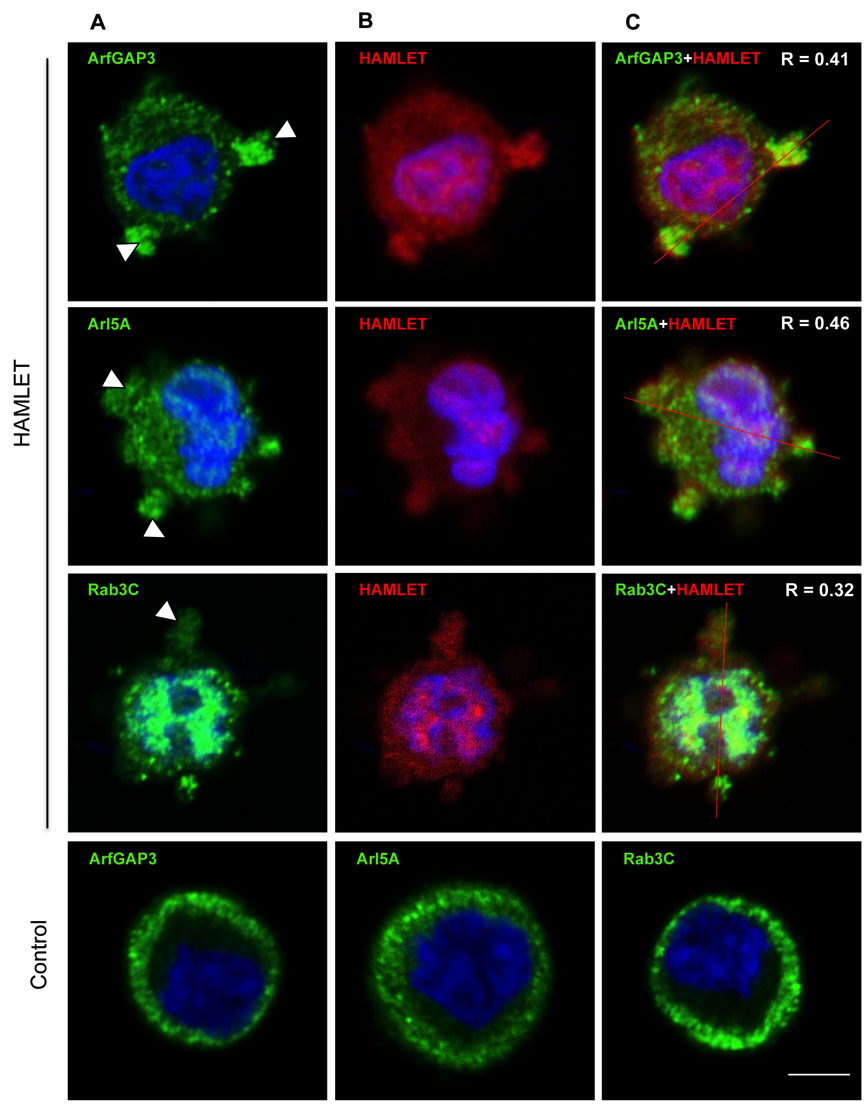
Supplementary Figure S5:** **Accumulation of Ras family proteins in membrane protrusions. (A)** Confocal images show the enrichment of different Ras proteins (green) (ArfGAP3, Arl5A and Rab3C) in tumor cell blebs compared to the cytoplasmic region.**(B)** HAMLET (red) was also present in the membrane blebs. **(C)** Merged confocal images. Red lines represent intensity distribution region used for calculating Pearson coefficient. R = Pearson coefficient for co-localization. Scale bar, 5 μm.

**
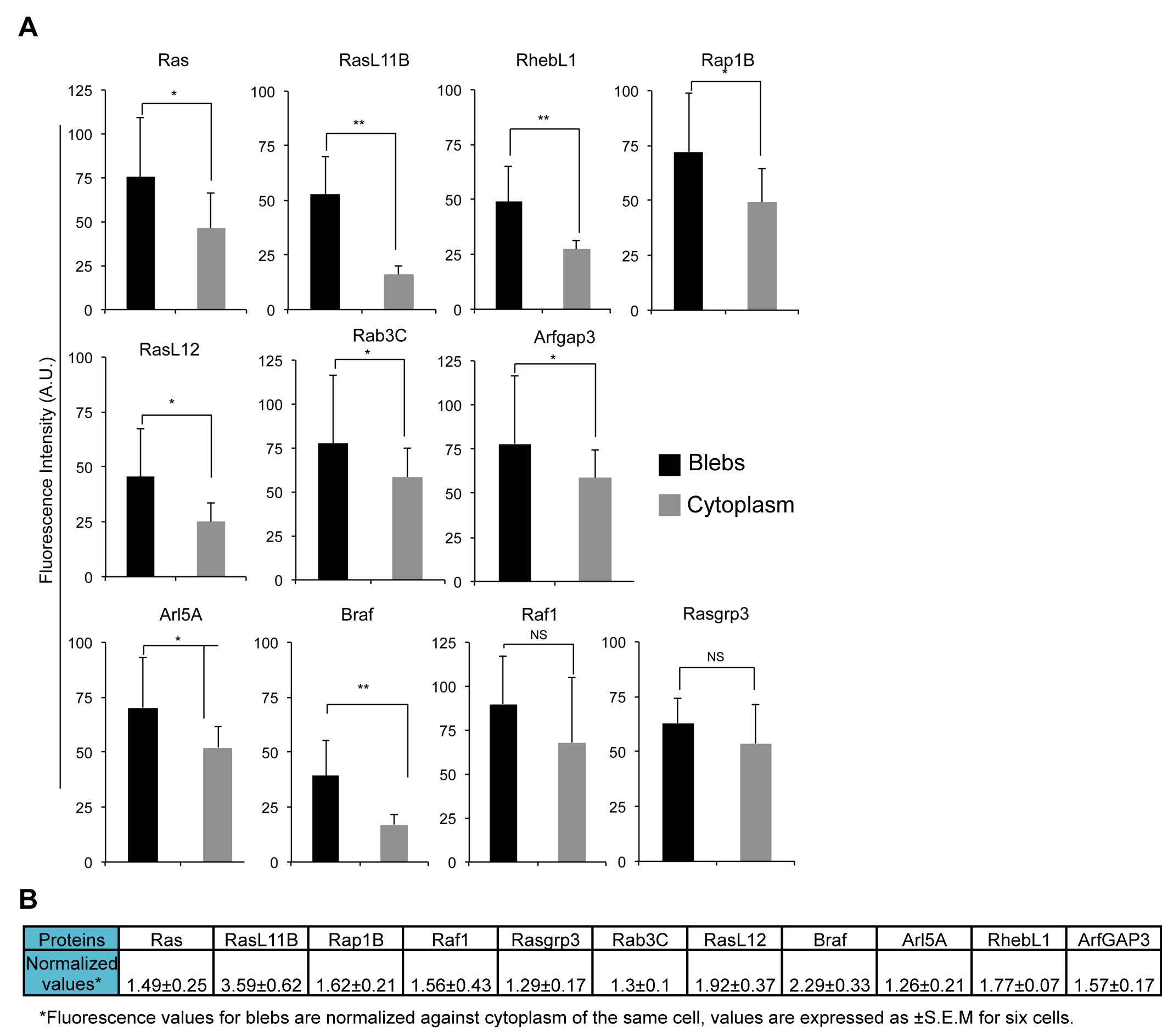
**

**Supplementary Figure S6: HAMLET and Ras proteins accumulates in tumor cell blebs. A)** Quantification of fluorescence intensity in the cytoplasm and blebs for Ras proteins shown in Fig. 4 and Supplementary Figure S4-S5. **p<0.01 and *p<0.05. **B)** Table shows normalized fluorescence values for blebs against cytoplasm of the same cell, values are expressed as ±S.E.M (n=6).

**
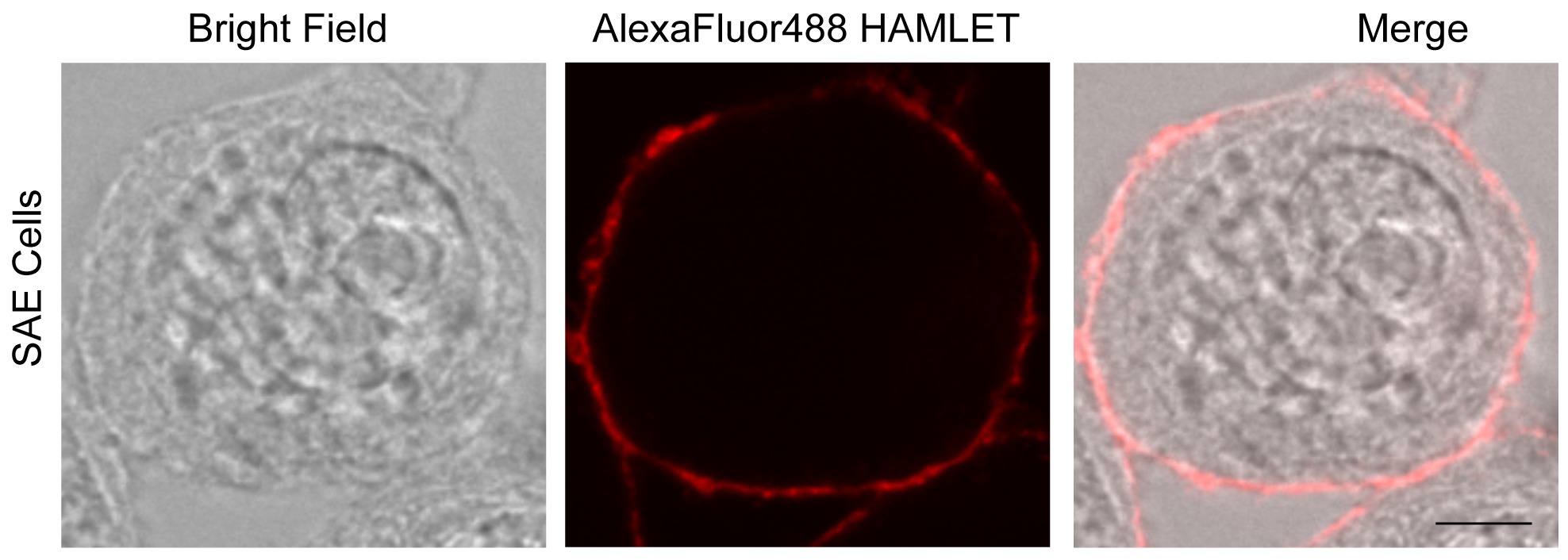
**

**Supplementary Figure S7: HAMLET interacts with the membrane of primary SAE cells.** Primary SAE cells were treated with Alexa-488 labeled HAMLET (red) at 4°C. The cell membrane binding of HAMLET was investigated under confocal microscope. Scale bar, 5 μm

**
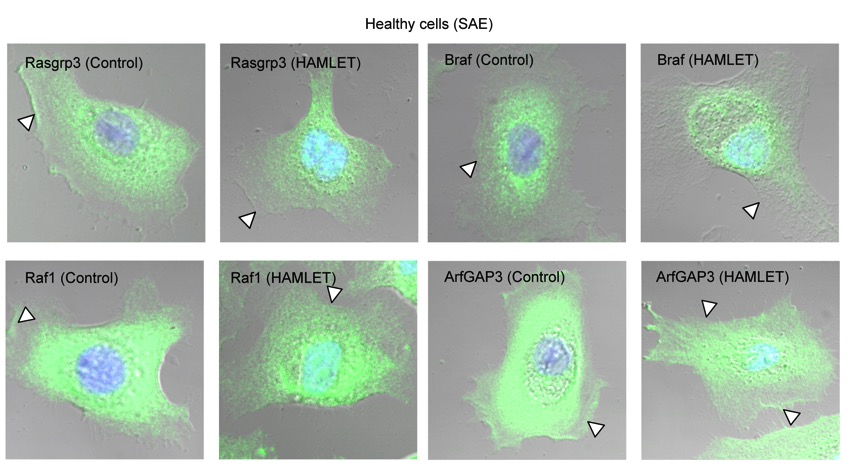
 Supplementary Figure S8: Localization of Ras proteins in healthy cells after HAMLET exposure.** Relocalization of Rasgrp3, Braf, Raf1 and ArfGAP3 from the plasma membrane to the cytoplasm in healthy SAE cells (30 minutes of HAMLET exposure). Scale bar 10 μm.

**
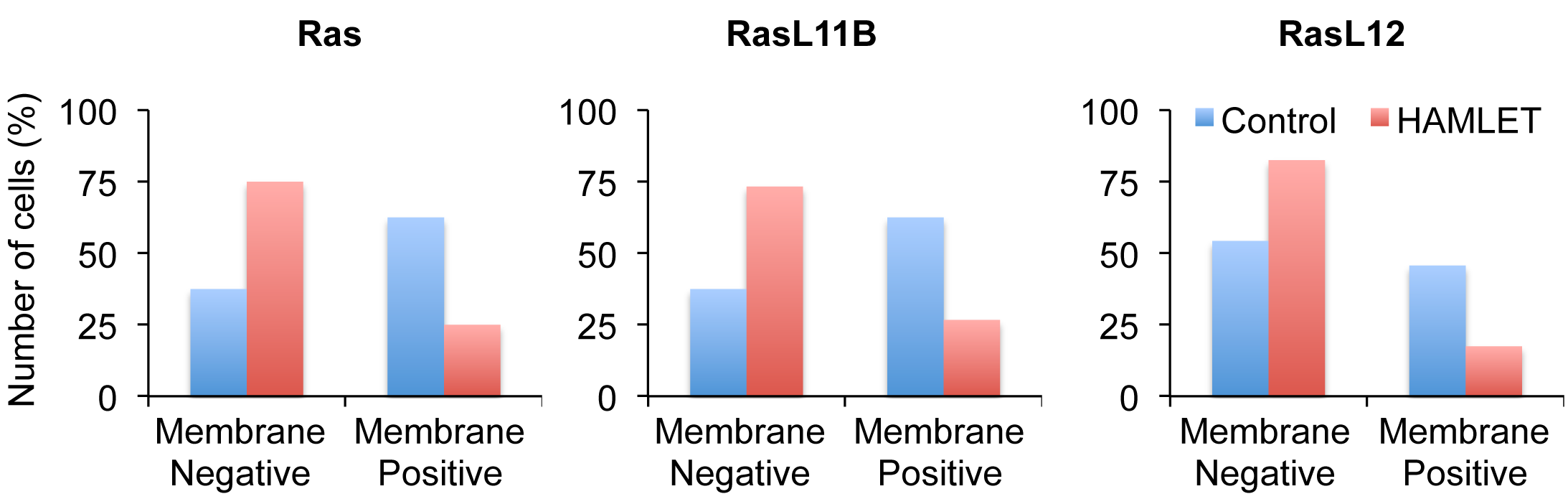
**

**Supplementary Figure S9: Loss of membrane staining for Ras, RasL11B and RasL12 staining from primary SAE cells.** Primary SAE cells treated with HAMLET showed loss of membrane staining for individual Ras proteins compare to untreated control. Data in graphs represents quantification of membrane positive and membrane negative cells (n=35).

**Supplementary Movie S1A:** **Interaction of unlabeled HAMLET with GUV model membranes.**

**Movie Caption:** Movie assembled from time-lapse spinning disk confocal fluorescence microscopy images (frame rate, 10 fps; total duration, 25 s; image size, 108 µm x 108 µm; scale bar, 40 μm). obtained for a population of electroformed GUVs (0.01 mg/mL total lipid concentration) consisting of POPC doped with 2% Rhodamine-B labeled DOPE membrane in an isotonically balanced sucrose solution (300 mM). Images were acquired immediately after administering 30 μM HAMLET in DPBS. Rhodamine B was exposed with a 50mW 561 laser line close to its excitation peak of 560nm and emitted at its peak of 583nm.

**Supplementary Movie S1B:** **Interaction of unlabeled HAMLET with GUV model membranes.**

**Movie Caption:**Movie assembled from time-lapse images of spinning disc confocal fluorescence microscopy images (frame rate, 1 fps; Total duration, 262s; Image Size, 170umx170um; Scale Bar, 40 μm) obtained for a population of electroformed GUVs (0.01 mg/mL total lipid concentration) consisting of POPC doped with 2% Rhodamine-B labeled DOPE membrane in an isotonically balanced sucrose solution (300 mM). Images were acquired immediately after administering 20 μM HAMLET in DPBS. Rhodamine B was exposed with a 50mW 561 laser line close to its excitation peak of 560nm and emitted at its peak of 583nm.

**Supplementary Movie S1C:** **Interaction of unlabeled HAMLET with GUV model membranes.**

**Movie Caption:** Movie assembled from time-lapse spinning disk confocal fluorescence microscopy images (frame rate, 10 fps; total duration, 33 s; image size, 170 µm x 170 µm; scale bar, 40 μm). obtained for a population of electroformed GUVs (0.01 mg/mL total lipid concentration) consisting of POPC doped with 2% Rhodamine-B labeled DOPE membrane in an isotonically balanced sucrose solution (300 mM). Images were acquired immediately after administering 30 μM HAMLET in DPBS. Rhodamine B was exposed with a 50mW 561 laser line close to its excitation peak of 560nm and emitted at its peak of 583nm.

**Supplementary Movie S2:** **Interaction of human α-lactalbumin (HLA) with GUV model membranes.**

**Movie Caption:** Movie assembled from time-lapse spinning disk confocal fluorescence microscopy images (frame rate, 10 fps; exposure, 20 ms; total duration,283 s; image size, 170 μm x 170 μm; scale bar, 30 μm) obtained for a population of electroformed GUVs (0.01 mg/mL total lipid concentration) consisting of POPC doped with 2% Rhodamine-B labeled DOPE membrane in an isotonically balanced sucrose solution (300 mM). Images were acquired immediately after administering 30 μM HLA in DPBS.

**Supplementary Movie S3: Interaction of oleic acid with GUV model membranes.**

**Movie Caption:** Movie assembled from time-lapse spinning disk confocal fluorescence microscopy images (frame rate, 12 fps; exposure, 32 ms; total duration, 78s; image size, 170 μm x 170 μm; scale bar, 30 μm) obtained for a population of electroformed GUVs (0.01 mg/mL total lipid concentration) consisting of POPC doped with 2% Rhodamine-B labeled DOPE membrane in an isotonically balanced sucrose solution (300 mM). Images were acquired immediately after administering 500 μM oleic acid.

**Supplementary Movie S4:** **Interaction of sodium oleate with GUV model membranes.**

**Movie Caption:** Movie assembled from time-lapse images of spinning disk confocal fluorescence microscopy images (frame rate, 10 fps; exposure, 20 ms; total duration, 495 s; image size, 170 μm x 170 μm; scale bar, 30 μm) obtained for a population of electroformed GUVs (0.01 mg/mL total lipid concentration) consisting of POPC doped with 2% Rhodamine-B labeled DOPE membrane in an isotonically balanced sucrose solution (300 mM). Images were acquired immediately after administering 500 μM sodium oleate

**Supplementary Movie S5:** **Interaction of Alexa Fluor-labeled HAMLET with GUV model membranes.**

**Movie Caption:** Movie assembled from time-lapse spinning disk confocal fluorescence microscopy images (frame rate, 0.1 fps; total duration, 220 s; image size, 80 μm x 66 μm; scale bar, 20 μm) obtained for a population of electroformed GUVs (0.01 mg/mL total lipid concentration) consisting of POPC doped with 3% Oregon Green 488 labeled DHPE membrane in an isotonically balanced sucrose solution (300 mM). Images were acquired immediately after administering 30 μM, 5% fluorescently conjugated Alexa Fluor 568 HAMLET solution.

**Supplementary Movie S6:** **Interaction of Alexa Fluor-labeled HAMLET with GUV model membranes.**

**Movie Caption:** Movie assembled from time-lapse spinning disk confocal fluorescence microscopy images (frame rate, 1 fps; total duration, 119 s; image size, 170 μm x 170 μm; scale bar, 20 μm) obtained for a population of electroformed GUVs (0.01 mg/mL total lipid concentration) consisting of POPC doped with 3% Oregon Green 488 labeled DHPE membrane in an isotonically balanced sucrose solution (300mM). Images were acquired immediately after administering 30 μM, 5% fluorescently conjugated Alexa Fluor 568 labeled HAMLET solution.

**Supplementary Movie S7:** **Interaction of Alexa Fluor-labeled HAMLET with GUV model membranes.**

**Movie Caption:** Movie assembled from time-lapse spinning disk confocal fluorescence microscopy images (frame rate, 1 fps; total duration, 48 s; image size, 170 μm x 170 μm; scale bar, 40 μm) obtained for a population of electroformed GUVs (0.01 mg/mL total lipid concentration) consisting of POPC doped with 3% Oregon Green 488 DHPE labeled probe isotonic in 300 mM sucrose. Images were acquired immediately after administering 30 μM, 5% fluorescently conjugated Alexa Fluor 568 HAMLET solution.

**Supplementary Movie S8:** **Interaction of unlabeled HAMLET with tumor cell lipid extract GUV membranes.**

**Movie Caption:** Movie assembled from time-lapse spinning disk confocal fluorescence microscopy images (frame rate, 2 fps; total duration, 26 s; image size, 108 µm x 108 µm; scale bar, 30 μm). GUVs were created from TCLE with Rhodamine-B DOPE labeled lipids in 98 and 2 percent by mass, respectively, by electroformation in 300mM Sucrose. Rhodamine B was exposed with a 50mW 561 laser line close to its excitation peak of 560nm and emitted at its peak of 583nm. Images were acquired immediately after administering 30 μM HLA in DPBS.

**Supplementary Movie S9:** **Interaction of HAMLET with A549 lung carcinoma cells.**

**Movie Caption:** Movie assembled from live confocal microscopy images (frame rate, 2 fps; total duration, 12 s; image size, 90 μm x 90 μm; scale bar, 20 μm) obtained for a population of tumor cells in RPMI-1640 medium. Live images were acquired before and after administering 35 μM HAMLET solution.
